# THE ASSOCIATIONS BETWEEN DAILY MEMORY LAPSES, FAMILY RELATIONSHIPS, AND DAILY AFFECT IN MIDDLE-AGED AND OLDER ADULTS

**DOI:** 10.1093/geroni/igae098.0510

**Published:** 2024-12-31

**Authors:** Heejung Jang, Nikki Hill, Jennifer Turner, David Almeida, Jacqueline Mogle

**Affiliations:** Clemson University, Clemson, South Carolina, United States; The Pennsylvania State University, University Park, Pennsylvania, United States; University of Hawaii Hilo, Hilo, Hawaii, United States; Pennsylvania State University, University Park, Pennsylvania, United States; Clemson University, Clemson, South Carolina, United States

## Abstract

Daily memory lapses represent an understudied approach to understanding daily experiences of cognitive functioning. Because family relationships greatly influence individuals’ daily lives, the present study explores how the quality of family relationships impact daily memory lapses and the moderating influence of family relationship quality on the link between daily memory lapses and daily affect. We used data from 1,236 middle-aged and older adults (Mage= 62.48 years, SD= 10.21, range 43-91; 57% female) drawn from the third waves of the Midlife in the United States and National Studies of Daily Experiences. Participants completed 8 nightly telephone diaries that included both prospective and retrospective memory lapses as well as daily positive and negative affect. At baseline, participants reported on the emotional support they experienced with their family. Latent class analysis (LCA) models identified four family relationship types: pleasant, ambivalent, neutral, and unpleasant. Compared to pleasant relationships, ambivalent (b=.23, p<.05) and neutral (b=.35, p<.01) relationships are strongly associated with frequency of prospective memory lapses, but not retrospective lapses. Compared to pleasant relationships, ambivalent (b=.02, p<.05), neutral (b=.02, p<.05), and unpleasant (b=.07, p<.001) relationships are associated with increased negative affect on days with a retrospective lapse, but not on days a prospective lapse. This study contributes to the literature by revealing that family relationships influence cognitive problems individuals experience in their daily lives and how these cognitive problems may contribute to affective symptom load over time.

